# Recommendations from the ERAS® Society for standards for the development of enhanced recovery after surgery guidelines

**DOI:** 10.1002/bjs5.50238

**Published:** 2019-12-02

**Authors:** M. Brindle, G. Nelson, D. N. Lobo, O. Ljungqvist, U. O. Gustafsson

**Affiliations:** ^1^ Department of Surgery Alberta Children's Hospital Calgary Alberta Canada; ^2^ Department of Community Health Sciences Alberta Children's Hospital Calgary Alberta Canada; ^3^ Division of Gynecologic Oncology Tom Baker Cancer Centre Calgary Alberta Canada; ^4^ Gastrointestinal Surgery Nottingham Digestive Diseases Centre and National Institute for Health Research Nottingham Biomedical Research Centre, Nottingham University Hospitals NHS Trust and University of Nottingham, Queen's Medical Centre Nottingham UK; ^5^ Medical Research Council–*Versus* Arthritis Centre for Musculoskeletal Ageing Research, School of Life Sciences University of Nottingham, Queen's Medical Centre Nottingham UK; ^6^ Department of Surgery Örebro University and University Hospital Örebro Sweden; ^7^ Institute of Molecular Medicine and Surgery, Karolinska Institutet Stockholm Sweden; ^8^ Department of Surgery Danderyd Hospital Stockholm Sweden; ^9^ Department of Clinical Sciences Danderyd Hospital, Karolinska Institutet Stockholm Sweden

## Abstract

**Background:**

ERAS® Society guidelines are holistic, multidisciplinary tools designed to improve outcomes after surgery. The enhanced recovery after surgery (ERAS) approach was initially developed for colorectal surgery and has been implemented successfully across a large number of settings, resulting in improved patient outcomes. As the ERAS approach is increasingly being adopted worldwide and new guidelines are being generated for new populations, there is a need to define an ERAS® Society guideline and the methodology that should be followed in its development.

**Methods:**

The ERAS® Society recommended approach for developing new guidelines is based on the creation of multidisciplinary guideline development groups responsible for defining topics, planning the literature search, and assessing the quality of the evidence.

**Results:**

Clear definitions for the elements of an ERAS guideline involve multimodal and multidisciplinary approaches impacting on multiple patient outcomes. Recommended methodology for guideline development follows a rigorous approach with systematic identification and evaluation of evidence, and consensus‐based development of recommendations. Guidelines should then be evaluated and reviewed regularly to ensure that the best and most up‐to‐date evidence is used consistently to support surgical patients.

**Conclusion:**

There is a need for a standardized, evidence‐informed approach to both the development of new ERAS® Society guidelines, and the adaptation and revision of existing guidelines.

## Graphical abstract

This paper presents the ERAS® Society recommended approach for developing new guidelines. Elements that define an enhanced recovery after surgery (ERAS) guideline include a multimodal, multidisciplinary approach impacting on multiple patient outcomes. Recommended methodology for guideline development follows a rigorous approach with systematic identification and evaluation of evidence, and consensus‐based development of recommendations.

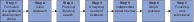

Clear guidance for guideline development

## Abstract (es)

**Antecedentes:**

Las guías de la sociedad ERAS® (*Enhanced Recovery After Surgery*) son herramientas holísticas y multidisciplinares diseñadas para mejorar los resultados después de la cirugía. Los programas ERAS (guías de recuperación intensificada) se desarrollaron inicialmente para la cirugía colorrectal y se han implementado con éxito en muchos otros ámbitos, lo que resulta en mejores resultados para los pacientes. A medida que los programas ERAS se adoptan cada vez más en todo el mundo y se generan nuevas guías para nuevas poblaciones, es necesario definir una guía clínica de la sociedad ERAS® y la metodología a seguir para su desarrollo.

**Métodos:**

La sociedad ERAS® recomienda que el enfoque para desarrollar las nuevas guías se base en el establecimiento de grupos multidisciplinares responsables de la definición de los temas, planteamiento de la revisión de la literatura y valoración de la calidad de la evidencia.

**Resultados:**

Las definiciones precisas de los elementos de una guía ERAS implican enfoques multimodales y multidisciplinares que tengan en cuenta los múltiples resultados que afectan a los pacientes. La metodología recomendada para el desarrollo de guías debe seguir un enfoque riguroso con identificación sistemática y evaluación de evidencia, y el desarrollo de recomendaciones basadas en el consenso. Posteriormente, las guías deben evaluarse y revisarse regularmente para garantizar que la evidencia mejor y más actualizada se aplique al manejo de los pacientes quirúrgicos.

**Conclusión:**

Es necesario un enfoque estandarizado, basado en la evidencia, tanto para el desarrollo de nuevas guías de la sociedad ERAS® como para la adaptación y revisión de las guías ya existentes.

## Introduction

Enhanced Recovery After Surgery (ERAS®) Society consensus statements and guidelines are powerful tools that have been implemented across hospitals and healthcare systems worldwide to improve the quality of surgical care. The ERAS approach was initially conceived by a group of surgeons in northern Europe, based on the principle that actions undertaken to modulate postoperative stress can improve recovery after surgery. The ERAS approach has shown that early mobilization, early reintroduction of nutrition, and rapid discharge are feasible and beneficial for a large number of postoperative patients^1^.

The first ERAS consensus paper was produced for colonic surgery in 2005^2^. Its expansion to other settings and countries resulted in multiple studies showing benefits in patients undergoing colorectal surgery^3^. Subsequent guidelines were developed using modifications of the colorectal ERAS approach and applied to various other surgical specialties, with excellent results^4^, ^5^, ^6^.

The initial ERAS consensus paper^2^ was developed by a team of expert clinical researchers and translated an understanding of physiological responses to surgical stress into clinical practice, with a focus on the specific needs of adults undergoing colorectal surgery. The guideline was produced by experts, based on a joint review of the literature and the quality of evidence supporting individual aspects of the guideline.

The strong principles underlying the ERAS concept, including its multidisciplinary and multimodal approach to perioperative care, and results of ERAS implementation may all have contributed to the success and wide adoption of the initial guideline in improving patient outcomes. Subsequent iterations and adaptations of the initial ERAS guideline have been produced using various methodologies, but have frequently used the initial consensus paper^2^ as a framework^7^.

Since the initial introduction of ERAS nearly 20 years ago, publications on ERAS have increased, with more than 1300 published in the past 5 years. New guidelines are created with variable quality and rigour from a wide variety of authors.

As the ERAS approach is being adopted increasingly worldwide and new guidelines are being generated for new populations, there has been, and remains, a need to define more precisely what constitutes an ERAS® Society guideline and what methodology should be followed for development. The ERAS® Society recognizes that there is a real need for a standardized, evidence‐informed approach to both the adaptation and the revision of existing guidelines, in addition to the development of new guidelines. ERAS guidelines, with their focus on broad multimodal care across a spectrum of time points and involving multiple specialties, require an ERAS‐specific approach.

In December 2018, an ERAS® Society guideline steering group was formed to standardize and improve the guideline development process, in order to transform it into a regulatory framework that could be followed by all surgical specialties that plan to update or establish new ERAS® Society guidelines. This document outlines the ERAS® Society guideline for ERAS® Society guideline development. This includes the general principles of what differentiates an ERAS® Society guideline from other guidelines and the recommended methods for developing new ERAS® Society guidelines. This framework is presented as a detailed step‐by‐step manual, with comment and justification of the proposed development process.

## Methods

ERAS® Society guidelines can be differentiated from other clinical guidelines in a number of ways, including their holistic, multidisciplinary design, rigorous and broad literature review, and strong grounding in ERAS expertise (*Table* 1).

**Table 1:** Requirements of ERAS® Society guidelines

ERAS guidelines target specific surgical procedures or a group of similar surgical procedures
ERAS guidelines are multidisciplinary and multiprofessional
ERAS guidelines should be developed by individuals from different health settings and different professions, with consideration for patient involvement
ERAS guidelines are holistic and should address elements of preoperative, intraoperative and postoperative care
ERAS guidelines address multiple patient outcomes
ERAS guidelines require endorsement from ERAS® Society leadership
Creation of ERAS guidelines should follow ERAS® Society methods
ERAS guidelines should be presented, when possible, using ERAS formatting, including an ERAS diagram
ERAS guidelines should be created with a plan for implementation, audit and evaluation

### Timeline of the guideline development process

To ensure that future guidelines are up to date with a rapidly moving field of research, a clear time frame for completion should be established. The recommended time span used for the development of new guidelines should be as short as is feasible, with most guidelines completed within 10–12 months.

### Guideline development process

#### *Step 1: Formation of a guideline development group*

When developing new guidelines, the ERAS® Society will work with lead authors to establish a guideline development group (GDG) that has international representation and will drive the process of guideline development. One or two lead authors will assume first and/or last author position on the published guideline and be responsible for ensuring the process is completed appropriately in a timely fashion. The number of members in an ERAS GDG should normally not exceed ten individuals in total, and should include two or three surgeons from the specialty under study and two anaesthetists and potentially other experts (see below). Input from patients and a diverse number of stakeholders is an important aspect of guideline development. Members of the GDG should be familiar with research methods and critical evaluation, whereas participation of other members can be included within the consensus generation process (see step 5) or in consultation for particular aspects of the guideline. At least one member of each GDG must have in‐depth knowledge of epidemiology and/or statistics to be able to judge the scientific quality of new and existing research adequately. All members of the GDG will be jointly responsible for the recommendations and co‐author the new guideline.

GDG members and expert consultants should include individuals with specialist knowledge in their field who can evaluate available evidence and understand the aspects of implementation when considering the value of specific recommendations. Specialist knowledge may accrue from recognized qualifications, the study of evidenced research, or clinical experience.

Patients and caregivers provide important perspectives on the appropriateness and acceptability of guidelines for patients and families^8^. Patient involvement should be meaningful, with patients having an opportunity to identify priorities during initial guideline generation and reflect on the feasibility and acceptance of final recommendations. Experts are defined as those recognized by their peers as having appropriate training and/or experience in their field, with practical and extensive knowledge of current and relevant issues, and who have a leadership role within their communities or societies.

Experts involved in guideline development could include: relevant healthcare specialists (such as surgeons, anaesthetists, oncologists, medical specialists, nurses, nutritionists), representatives of professional bodies, including national organizations, researchers (such as epidemiologists, statisticians) and health economists, as well as patients.

#### *Step 2: Establishing guideline topics and initial approach*

ERAS® Society guidelines may be adapted from existing guidelines or created *de novo*. When appropriate, existing guidelines may provide a framework for the development of a new guideline. If this process is followed, each element adapted from the previous guideline must be assessed individually for appropriateness for the new patient population for which the guideline is being created. This assessment should take the form of a series of focused literature reviews, with assessment of the quality of the evidence and development of recommendations. Recommendations should be specific to the specialty with elements added, removed or edited based on the literature review. The GDG should have a process whereby new relevant elements can be identified for inclusion. This may take the form of a formal Delphi process^9^ or other method of consensus generation.

ERAS guidelines can be developed *de novo* for unique populations. Methods to develop proposed guideline elements should take the form of a Delphi process, with development of a large number of proposed topics or elements that are voted on for inclusion by the GDG and informed by existing relevant guidelines. The process of focused literature reviews should be followed for each proposed recommendation.

#### *Step 3: Scoping the guideline and planning the literature search*

The first task for the GDG is to scope the guideline, and plan and carry out the relevant literature search. Relevant existing guidelines and priority areas should be identified^10^. The scoping process subsequently informs the targeted literature searches performed by the GDG, and therefore involves the process of setting key inclusion and exclusion criteria, and identifying target outcomes.

If the GDG does not have expertise in systematic reviews or knowledge syntheses, this should be done in consultation with a research librarian or other knowledge synthesis expert. The PICO (Population, Intervention, Comparator and Outcome) framework^11^, ^12^, ^13^ should be used to help formulate clear review questions and aid a systematic review of the evidence (*Table* 2). The GDG should identify all relevant databases, registries, audits and surveys to be used for the literature search, as well as important key words and search terms within certain date and language restrictions. Unlike traditional systematic reviews, the search strategy should be focused and supplemented by citation searching and expert identification of relevant papers to ensure a feasible screening process. The goal of the search strategy is not to obtain a comprehensive summary of the literature, but rather to ensure that the most relevant information is acquired. As a rule, all available evidence for each single ERAS intervention should be captured in the literature search for later assessment. The search strategy should be recorded within appendices and be transparent to allow repetitive search sessions and reviews by two independent experts, external to the GDG.

**Table 2:** PICO (Population, Intervention, Comparator and Outcome) framework

Population	Which patient population is being studied?
Intervention	Which treatment or intervention is being recommended?
Comparator	Which alternative treatments are available?
Outcome	Which end points are being studied?

Unlike a systematic review, screening titles, abstracts and full texts for the multiple searches can be performed by a single individual, but the final body of literature should be reviewed by the GDG to ensure that relevant papers familiar to the other group members are captured. The screening process for each question and each search should be recorded within a PRISMA^14^ diagram.

#### *Step 4: Analysing the quality of evidence*

All studies captured in the literature search should undergo a standardized process of evaluation, regardless of study design. Each single intervention in the ERAS protocol should be scrutinized, and quality assessed according to the Grading of Recommendations, Assessment, Development and Evaluation (GRADE) approach^15^. This means that a GRADE assessment form with a final rating of the quality of the evidence should be provided for every study reviewed. The final number of studies to be included in the table of evidence is decided upon by the GDG, depending on the quality of evidence supporting each ERAS intervention.

The GRADE assessment approach provides a structured way to consider key factors that may increase or decrease confidence in a synthesized body of evidence. In contrast to alternative grading systems, GRADE is used to grade the quality of evidence in the body of literature supporting the evidence for the relationship between interventions and outcomes, rather than of the individual studies *per se* ^15^. Therefore, the quality of evidence provided by the studies included in the final analysis should be classified as high, moderate, low and very low by assessing the following aspects: importance of outcomes, risk of bias, heterogeneity, indirectness, imprecision and publication bias (*Table* 3).

**Table 3:** GRADE assessment of evidence^15^

Assigned GRADE quality	Description
High	Further research is very unlikely to change confidence in the estimate of effect
Moderate	Further research is likely to have an important impact on confidence in the estimate of effect and may change the estimate
Low	Further research is very likely to have an important impact on confidence in the estimate of effect and is likely to change the estimate
Very low	Any estimate of effect is very uncertain

GRADE, Grading of Recommendations, Assessment, Development and Evaluation.

After a GRADE assessment of the evidence, a recommendation should follow, rating the strength of the recommendation as either strong or weak based on a number of influential factors, including the GRADE quality of the evidence (*Table* 4). In the setting of low or very low evidence, there is significant burden on the GDG to defend a strong recommendation. In this evaluation, the magnitude of effect, cost‐effectiveness and expected treatment burden for the patient should be considered^15^, ^16^.

**Table 4:** GRADE assessment of strength of recommendations^15^

Assigned GRADE strength of recommendation	Description
Strong	Desirable effects of intervention clearly outweigh undesirable effects, or clearly do not
Weak	Trade‐offs are less certain, either because of low‐quality evidence or because evidence suggests desirable and undesirable effects are closely balanced

GRADE, Grading of Recommendations, Assessment, Development and Evaluation.

#### *Step 5: Review by two independent experts*

Once the process of literature search, scoring of each study, GRADE assessment of evidence and recommendation has been completed by the GDG group, all steps should be reviewed by two independent experts appointed by the ERAS® Society. These two experts must approve the process before it can be considered to be complete, and they will also be jointly responsible for the recommendations and participate as guideline co‐authors.

#### *Step 6: Resolution of disagreement and consensus generation*

The GDG and the two independent experts will identify recommendations that have been particularly difficult to decide upon. This situation may arise in the setting of evidence that is difficult to interpret or translate into recommendations, or recommendations that may be controversial or radically alter previous knowledge. To make sure such complicated questions are dealt with in an optimal manner, they should also be reviewed in a Delphi process or other method of consensus generation. This process should involve an expanded panel of no more than ten relevant experts appointed by the ERAS® Society (who will be listed in the acknowledgements of the final guidelines).

The Delphi process is one recommended method to achieve expert consensus^17^. Within this aspect of guideline generation, the most complicated or controversial issues will be summarized and distributed in the form of structured questionnaires to the panel of experts, who will answer questions anonymously, weight and justify their responses. The process may undergo several rounds, to encourage the panel to attain consensus.

#### *Step 7: Finalizing the process and submitting new guidelines*

**Figure 1:**
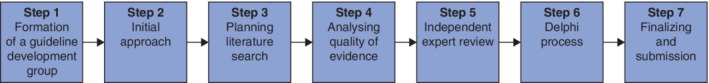
Process of generation of an ERAS® Society guideline

When all of the above steps have been completed (*Fig*. 1), the final draft must be approved by all co‐authors before submission to a peer‐reviewed journal. Agreements with the journals must be made before submission to secure that all guidelines produced by the ERAS® Society are made available for free or open‐access download via the ERAS® Society website.

**Figure 2:**
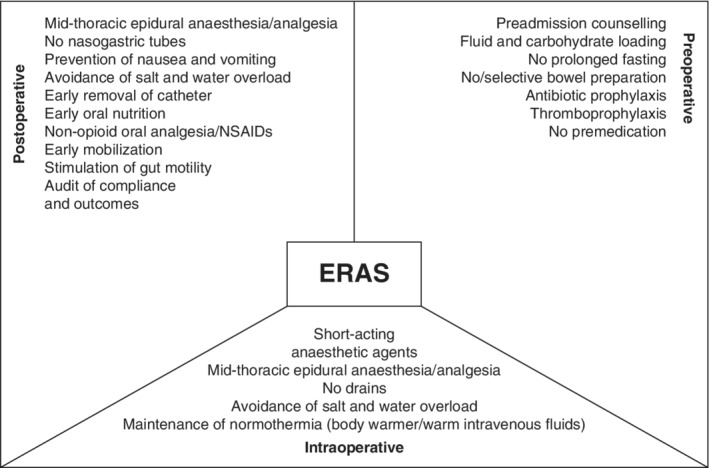
Example of an ERAS® Society diagram ERAS, enhanced recovery after surgery; NSAID, non‐steroidal anti‐inflammatory drug. (Redrawn from Varadhan *et al*.^18^, with permission.)

The format of the guideline should adhere to the guidelines of the scientific journal, but should include a clear description of the methods outlined above, including the identification of proposed elements, the literature search, the rating of quality, the generation of final recommendations, and the rating of the strength of those recommendations. Results should include an ERAS® Society diagram (*Fig*. 2)^18^ containing elements within the preoperative, intraoperative and postoperative period. Each ERAS element should be accompanied by a short narrative; an evidence table (or a table included within the appendices) and the GRADE quality of evidence and strength of recommendation should be provided.

### Revising guidelines

Every 2–3 years, the lead author or designated alternative will present a formal report on the guideline to the ERAS® Society, with a brief re‐evaluation of the literature. If there is substantial new information, a guideline update will be performed. This review should be a facilitated process, generally lasting no more than 3–4 months. Relevant recommendations should have an updated search performed to include papers published since the last guideline. Inclusion of the recommendation, its wording, the quality of evidence and strength of recommendations should be considered in light of new evidence. The GDG should also consider whether new recommendations should be added to the guideline. As with creation of a new guideline, the revised guideline should be reviewed by external reviewers identified by the ERAS® Society. In the case of a simple revision without substantial changes (no changes to the recommendations themselves), a single reviewer would suffice. In the case of revised recommendations, or if recommendations are eliminated or added, two reviewers and potentially the Delphi process would be advised.

## Discussion

Since 2005, 23 ERAS® Society publications of 20 guidelines have been published for surgery and perioperative care in various specialties and subspecialties^19^ (*Table* *S1*, supporting information)^2^, ^7^, ^20^, ^21^, ^22^, ^23^, ^24^, ^25^, ^26^, ^27^, ^28^, ^29^, ^30^, ^31^, ^32^, ^33^, ^34^, ^35^, ^36^, ^37^, ^38^, ^39^, ^40^. The methodology described above ensures consistency in the development of guidelines that, in turn, can be used and updated continuously to inform perioperative care across multiple surgical specialties.

## Disclosure

All authors are members of the ERAS® Society.

## Supporting information


**Appendix S1.** Supporting information
