# Supplementary material for: Recommendations from the ERAS® Society for standards for the development of enhanced recovery after surgery guidelines
Source: BJS Open. 2019 Dec 2;4(1):157–63. doi: 10.1002/bjs5.50238 (PMC6996628; doi:10.1002/bjs5.50238)
Supplement: Supplementary file 1 — Appendix S1. Supporting information [file BJS5-4-157-s001.docx]

**BJS5_50238**

**Recommendations from the ERAS^®^ Society for standards for the development of enhanced recovery after surgery guidelines**

**M. Brindle, G. Nelson, D. N. Lobo, O. Ljungqvist and U. O. Gustafsson**

**Table S1** ERAS® Society guidelines published in peer-reviewed journals^19^

|  | Procedures/Specialty | Lead author | Reference | No. of citations** (19 August 2019) |
| --- | --- | --- | --- | --- |
| 1 | Colonic resection | Fearon KC | Clin Nutr. 2005;24:466-77 | 648 |
| 2 | Colorectal surgery | Lassen K | Arch Surg. 2009;144:961-9 | 570 |
| 3* | Pancreaticoduodenectomy | Lassen K | Clin Nutr. 2012;31:817-30 | 216 |
| 4* | Pancreaticoduodenectomy | Lassen K | World J Surg. 2013;37:240-58 | 158 |
| 5* | Colonic surgery | Gustafsson UO | Clin Nutr. 2012;31:783-800 | 346 |
| 6* | Colonic surgery | Gustafsson UO | World J Surg. 2013;37:259-84 | 514 |
| 7* | Rectal/pelvic surgery | Nygren J | Clin Nutr. 2012;31:801-16 | 192 |
| 8* | Rectal/pelvic surgery | Nygren J | World J Surg. 2013;37:285-305 | 214 |
| 9 | Radical cystectomy | Cerantola Y | Clin Nutr. 2013;32:879-87 | 206 |
| 10 | Gastrectomy | Mortensen K | Br J Surg. 2014;101:1209-29 | 196 |
| 11 | Anaesthesia for gastrointestinal surgery (Part I) | Scott MJ | Acta Anaesthesiol Scand. 2015;59:1212-31 | 77 |
| 12 | Anaesthesia for gastrointestinal surgery (Part II) | Feldheiser A | Acta Anaesthesiol Scand. 2016;60:289-334 | 142 |
| 13 | Gynaecologic oncology (Part I) | Nelson G | Gynecol Oncol. 2016;140:313-22 | 116 |
| 14 | Gynaecologic oncology (Part II) | Nelson G | Gynecol Oncol. 2016;140:323-32 | 108 |
| 15 | Bariatric surgery | Thorell A | World J Surg. 2016;40:2065-83 | 78 |
| 16 | Liver surgery | Melloul E | World J Surg. 2016;40:2425-40 | 99 |
| 17 | Breast reconstruction | Temple-Oberle C | Plast Reconstr Surg. 2017;139:1056e-1071e | 36 |
| 18 | Reporting of results | Elias KM | World J Surg. 2019;43:1-8 | 3 |
| 19 | Oesophagectomy | Low DE | World J Surg. 2019;43:299-330 | 8 |
| 20 | Lung surgery | Batchelor TJ | Eur J Cardiothorac Surg. 2019;55:91-115 | 12 |
| 21 | Colorectal surgery | Gustafsson UO | World J Surg. 2019;43:659-95 | 7 |
| 22 | Gynaecologic oncology | Nelson G | Int J Gynecol Cancer. 2019;29:651-68 | 2 |
| 23 | Cardiac surgery | Engelman DT | JAMA Surg 2019 May 4. [Epub ahead of print] doi: 10.1001/jamasurg.2019.1153 | 0 |

*Published simultaneously in Clin Nutr and World J Surg

** From Web of Science
